# The Effect of Shear Force on Skin Viability in Patients with Type 2 Diabetes

**DOI:** 10.1155/2019/1973704

**Published:** 2019-11-04

**Authors:** Luuk A. de Wert, Margot Geerts, Sander van der Brug, Laura Adriaansen, Martijn Poeze, Nicolaas Schaper, Nicole D. Bouvy

**Affiliations:** ^1^Department of General Surgery, Maastricht University Medical Centre, Maastricht, Netherlands; ^2^NUTRIM School for Nutrition and Translational Research in Metabolism, Maastricht University, Maastricht, Netherlands; ^3^Department of Neurology, Maastricht University Medical Centre, Maastricht, Netherlands; ^4^Department of Internal Medicine, Maastricht University Medical Centre, Maastricht, Netherlands; ^5^CARIM School for Cardiovascular Diseases, Maastricht University, Maastricht, Netherlands; ^6^CAPHRI School for Public Health and Primary Care, Maastricht University, Maastricht, Netherlands

## Abstract

**Background:**

Shear is a major risk factor in the development of diabetic foot ulcers, but its effect on the skin of patients with type 2 diabetes mellitus (DM) remains to be elucidated. The aim was to determine skin responses to shear in DM patients with and without diabetic polyneuropathy (DNP).

**Methods:**

The forearm skin was loaded with 14.5 N shear (+2.4 kPa pressure) and with 3.5 kPa pressure for 30 minutes in 10 type 2 DM patients without DNP, 10 type 2 DM patients with DNP, and 10 healthy participants. A Sebutape collected IL-1*α* (measure of tissue damage). A laser Doppler flowmeter measured cutaneous blood cell flux (CBF) as a measure of the reactive hyperaemic skin response.

**Findings:**

Reactive hyperaemia and IL-1*α* release was significantly increased after shear loading in all three groups and was higher compared to the responses to pressure loading. The reactive hyperaemic response after shear loading was impaired in patients with type 2 DM compared to healthy participants but did not differ between patients with and without DNP. The reactive hyperaemic response was negatively correlated with the blood glucose level but did not correlate with the DNP severity score.

**Interpretation:**

Shear is important in the development of tissue damage, but the reparative responses to shear are impaired in patients with type 2 DM. DNP was not associated with altered skin responses, suggesting that the loss of protective sensation to sense shear to skin remains a key factor in the development of diabetic foot ulcers in patients with DNP.

## 1. Introduction

Poorly healing diabetic foot ulcers, one of the most feared complications of diabetes mellitus (DM), are usually caused by a close interplay of biomechanical, neurological, and frequently also vascular abnormalities [1]. In patients with diabetic neuropathy (DNP), structural and functional abnormalities in the foot, in combination with an altered gait, lead to increased plantar foot pressures and increased shear during walking. The harmful effects of elevated pressure on the skin and subcutaneous tissues are relatively well defined, and elevated plantar pressures predict future ulceration [2]. Several lines of evidence suggest that not only the biomechanical loading on the skin and subcutaneous structures is increased in these patients but also that several reparative processes are also impaired in diabetes [3]. Once local pressure is relieved, the skin microvasculature reacts with a hyperaemic response in order to increase local blood flow, which is important for tissue repair [4, 5]. The hyperaemic response of the forearm skin to pressure was in one study diminished in patients with type 2 diabetes, probably contributing to impaired tissue repair after mechanical stress [6]. In addition to pressure, elevated shear also plays an important role in causing tissue damage [7–9], but reliable measurement of shear, e.g., on the surface of the foot during walking, is a difficult challenge. However, local elevations in plantar shear are associated with callus in the foot [10], which in its turn is a marked risk factor for future ulceration in earlier studies [11, 12].

In healthy tissues, damage such as pressure and/or shear will elicit the release of “damage-associated molecular patterns (DAMPs),” endogenous molecules that play a pivotal role in the early steps of tissue repair [13]. Animal studies suggest that the release of these immunological signalling molecules after noxious stimuli is impaired in diabetes [3]. Well-known DAMPs in the epidermis are interleukin-1*α* (IL-1*α*), interleukin-33 (IL-33), human mobility group box-1 (HMGB1), ATP, and DNA strains [14]. IL-1*α* is an early marker of mechanically induced skin damage in the skin, because it is rapidly released from the keratinocytes when they are injured [15, 16] and can be used as a marker to assess the loaded skin status [17–19]. The effect of DM and DNP on DAMP production of the human skin in humans is, as far as we know, not well studied yet. Moreover, little is known about the responses of the diabetic skin to shear and the influence of neuropathy on this response.

Obtaining more insight in the microvascular and immunological response of the diabetic skin to shear could facilitate the development of more fundamental strategies in preventing foot ulceration and stimulating (early) wound healing. As previously described [7], we developed a model to apply shear in combination with pressure on the forearm in humans enabling us to study both the local reactive hyperaemic and immunological response of the skin to pressure and shear. The aim of this study was therefore to determine these responses to shear loading (combined with pressure) or pressure loading alone in healthy volunteers and patients with type 2 DM with and without DNP.

## 2. Methods

### 2.1. Ethical Considerations

The research protocol was approved by the medical ethics committee of the Maastricht University Medical Centre and registered in the clinicaltrials.gov database (number NCT02348294). The study was conducted in compliance with ethical rules for human experimentation that are stated in the Declaration of Helsinki and monitored according to the principles of Good Clinical Practice.

### 2.2. Participants

Thirty participants with an age between 40 and 75 years were invited to participate in this study. Participants were divided into three groups of ten participants each: healthy volunteers (control), patients with type 2 DM but without symmetrical sensorimotor polyneuropathy (DM2 PNP-), and patients with type 2 DM with symmetrical sensorimotor polyneuropathy (DM2 PNP+). The groups were matched for age, sex, and body mass index (BMI).

Patients with type 2 DM were recruited at the outpatient diabetes clinic of the Department of Internal Medicine of the Maastricht University Medical Centre. Written informed consent was obtained from all participants. Exclusion criteria included any active skin diseases (such as psoriasis and eczema), autoimmune diseases, liver or renal insufficiency, peripheral arterial diseases (ankle brachial index < 0.9), and a poor glycaemic control in the last three months (HbA1c > 11.0%). Participants were not allowed to use any corticosteroids or a nonsteroidal anti-inflammatory drug at least seven days before the experiment. In addition, participants were not allowed to smoke or drink caffeine and/or alcohol during the test day.

### 2.3. Physical Examination

The diagnosis of DNP was based on a standardised clinical neurological examination (CNE), and its presence as well its severity was assessed using a validated scoring system [20]. This clinical scoring system corresponds well with the results of the neurophysiological examination and has acceptable sensitivity and specificity for the diagnosis of DNP when a cut-off point > 4 is used [21]. A CNE score of 0-4 indicated the absence of DNP, 5-14 moderate DNP, and 15-33 severe DNP. The clinical examination consisted of assessment of lower extremity muscle strength (plantar flexion foot, dorsiflexion digitorum 1), Achilles tendon reflex, and sensory function of the feet and legs (vibration sense, pinprick sense, light touch sense, and position sense). Peripheral arterial disease was ruled out based on history (no complaints or previous vascular surgery), presence of all peripheral arterial pulses, and a Doppler ankle brachial index > 0.9.

### 2.4. Procedure

Participants were tested at room temperature after an acclimatisation period of 20 minutes. The borders of the test area (50 mm × 50 mm) were marked on the volar aspect of both forearms at two centimetres of the centre of the cubital fossa. Baseline measurements of the unloaded skin within the marked area were performed at both arms. First, a small adhesive patch of Sebutape (Cuderm Corp, Dallas, TX) was attached to the skin for two minutes to collect proteins from the skin. Sebutape is a validated and reliable technique designed to absorb sebum and proteins from the skin surface [22].

Subsequently, the area was scanned back and forth with a single-point laser beam (infrared 785 nm wavelength, MoorLDI2 Burn Imager, Moor Instruments Ltd, Axminster, United Kingdom) to create a cutaneous blood cell flux map. In this map, a region of interest (ROI) of 30 × 30 mm in the centre marked area was selected and the mean velocity of the red blood cells was calculated with the Moor software version 5.3 expressed in the cutaneous blood cell flux (CBF) in arbitrary units (AU).

Then, the participants were requested to put both forearms on support cushions and loading was applied for 30 minutes on the designated area on both arms at the same time; one arm was loaded with 3.5 kPa pressure (pressure alone); the other arm was loaded with 14.5 N shear (combined with 2.4 kPa pressure) [7]. This model, which enabled us to apply pressure and shear, was described in detail elsewhere [7].

Postload measurements with the Sebutape and laser Doppler were performed on both arms directly after loading and repeated after 5, 10, 15, 20, and 60 minutes. In each participant, the arm which received pressure and the arm which received shear (combined with pressure) were randomized.

### 2.5. Biochemical Analysis

The extraction of proteins from the Sebutape was based on a study by Perkins and colleagues [22]. Briefly, two millilitres (ml) of Phosphate-Buffered Saline was added to each Sebutape sample, sonicated for ten minutes and vortexed for two minutes.

IL-1*α*, EGF, and IL-33 concentrations in the samples were determined by commercially available enzyme-linked immunosorbent assay (ELISA) kits (DuoSet R&D Systems) with a detection range of 3.9-250 pg/ml for IL-1*α* and EGF and a detection range of 5.9-1500 pg/ml for IL-33. To determine IL-6 concentrations in our samples, ELISA with a detection range of 7.8-1000 pg/ml was used.

### 2.6. Statistics

Statistical analysis was performed using GraphPad Prism 5 (GraphPad Software Inc., San Diego, CA) for Windows 10. Normality was tested with the D'Agostino Pearson Omnibus test. Data are expressed as mean with standard deviation (SD) when normally distributed or as median + interquartile range (IQR) when not normally distributed.

The parametric paired *t*-test or nonparametric Wilcoxon signed rank test was used to determine significance between pairwise comparisons. The parametric one-way ANOVA test followed by a Bonferroni post hoc correction or the nonparametric Kruskal-Wallis test followed by a Dunn multiple comparison test was used to determine significance between the groups. The parametric Pearson *r* or the nonparametric Spearman rank test was used to determine the correlation between different variables. A *P* value less than 0.05 was considered statistically significant.

## 3. Results

All experiments were performed between April 2015 and January 2016. In total, 30 participants were included and every participant finished the test day without any dropouts reported. The clinical characteristics of the participants are presented in Table 1. In summary, we studied elderly participants with overweight and the three groups were well matched for age, sex, and BMI. Most participants in the DM PNP+ group had moderate polyneuropathy based on clinical neurological examination. Glycaemic control was suboptimal in most participants with DM with elevated HbA1c and elevated blood glucose levels in many during the experiment.

### 3.1. Cutaneous Blood Cell Flux

**Figure 1:**
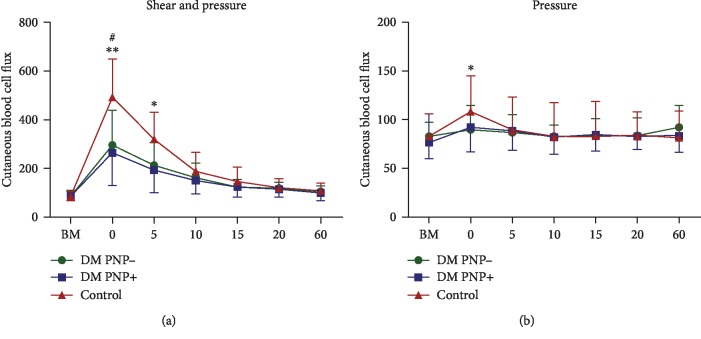
Cutaneous blood cell flux in arbitrary units (AU) presented as mean ± SD. BM indicates baseline measurement before loading. 0, 5, 10, 15, 20, and 60 indicate the time points after loading of the skin (in minutes). (a) Cutaneous blood cell flux measurements after the application of pressure and shear. ^∗^DM PNP+ vs. control (*P* < 0.05) at this time point. ^∗∗^DM PNP- vs. control (*P* < 0.05) and DM PNP+ vs. control (*P* < 0.01). ^#^Statistical significant value of *P* < 0.0001 in the control group, *P* < 0.001 in the DM PNP- group, and *P* < 0.01 compared to their own baseline measurements. (b) Cutaneous blood cell flux measurements after the application of pressure alone. ^∗^Statistical significant value of *P* < 0.05 at this time point of the DM PNP+ group or the control group compared with their own baseline measurement. No statistically significant differences were measured between the groups.

After the application of shear (combined with pressure) for 30 minutes, there was a statistically significant increase in CBF (reactive hyperaemic response) in all three groups (Figure 1(a)). In all three groups, the CBF (in arbitrary units (AU)) was significantly increased immediately after unloading (time point 0) compared to baseline: healthy group (mean 492.5 and SD 155.6 vs. mean 82.0 and SD 20.8, *P* < 0.0001), DM PNP- group (mean 296.2 and SD 142.7 vs. mean 91.8 and SD 19.1, *P* < 0.001) and DM PNP+ group (mean 264.8 and SD 134.2 vs. mean 89.5 and SD 20.2, *P* < 0.01).

In addition, this reactive hyperaemic response was 66.3% higher in the control group compared to the CBF of the DM PNP- group (*P* < 0.05) and 86.0% higher compared to the CBF of the DM PNP+ group (*P* < 0.01) (Figure 1(a)). No differences were observed in the reactive hyperaemic response between the DM patients with and without DNP. After 5 minutes, the CBF of the control group remained significantly higher compared with the CBF of the DM PNP+ group (*P* < 0.05). No statistically significant differences were observed between the groups at the other time points.

After the application of pressure alone, the CBF immediately after unloading (time point 0, in AU) was significantly higher compared to baseline in the control group (mean 109.5 and SD 36.8 vs. mean 84.1 and SD 22.7, *P* < 0.05) and that in the DM PNP+ group (mean 92.8 and SD 25.2 vs. mean 77.1 and SD 16.4, *P* < 0.05). In the DM PNP- group, the application of pressure alone did result in numerically higher CBF at time point 0, but this was not statistically significant (mean 90.4 and SD 25.1 vs. mean 83.5 and SD 14.8, *P* > 0.05). No statistically significant differences were observed in the postload reactive hyperaemic response between the three groups after the application of pressure alone (Figure 1(b)).

### 3.2. Cytokine Release

Immediately after unloading of the skin with shear (combined with pressure), the median skin IL-1*α* concentration (pg/ml) was increased compared to baseline in all three groups (control group 57.0 (29.5-167.1) vs. 19.1 (12.4-43.4) (*P* < 0.01), DM PNP- group 50.5 (25.6-143.4) vs. 9.7 (5.8-16.4) (*P* < 0.01), and DM PNP+ group 43.5 (21.3-61.6) vs. 15.5 (10.6-33.8) (*P* < 0.01)).

**Figure 2:**
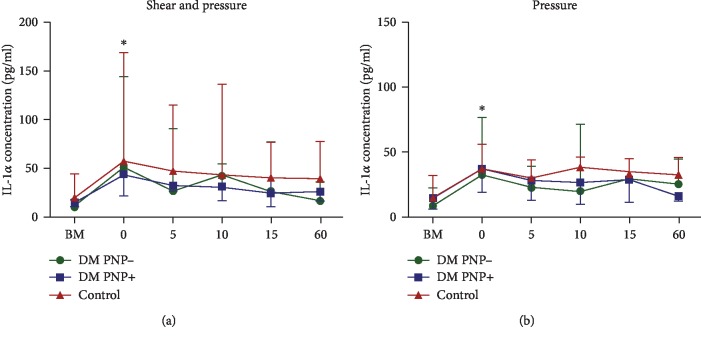
IL-1*α* concentration (pg/ml) presented as median and IQR. BM indicates baseline measurement before loading. 0, 5, 10, 15, and 60 indicate the time points after loading of the skin (in minutes). (a) IL-1*α* concentration measurements after the application of pressure and shear. ^∗^Statistical significant value of *P* < 0.01 in all three groups compared with their own baseline measurement. No statistically significant differences were measured between the groups. (b) IL-1*α* concentration measurements after the application of pressure and shear. ^∗^Statistical significant value of *P* < 0.01 in all three groups compared with their own baseline measurement.

**Figure 3:**
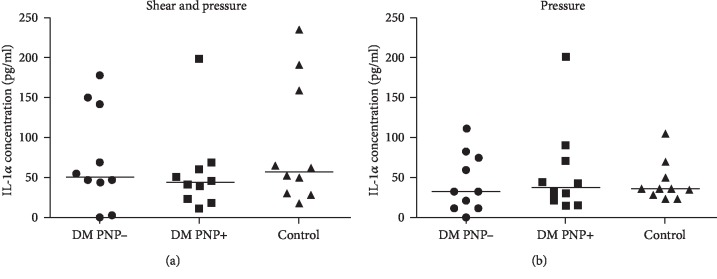
(a) IL-1*α* concentration directly after pressure and shear loading (time point 0). The bar indicates the median, while a dot represents the IL-1*α* concentration in one participant. No statistically significant differences were measured between the groups. (b) IL-1*α* concentration directly after pressure alone (time point 0). The bar indicates the median, while a dot represents the IL-1*α* concentration in one participant. No statistically significant differences were measured between the groups.

The same pattern was seen after the application of pressure alone, with an increase in the median IL-1*α* concentration in the control group 36.0 (26.8-55.4) vs. 15.4 (12.4-31.5) (*P* < 0.01), the DM PNP- group 32.2 (11.4-76.3) vs. 8.6 (0.8-21.9) (*P* < 0.01), and the DM PNP+ group 37.2 (19.5-76.6) vs. 14.5 (6.0-36.1) (*P* < 0.01). No statistically significant differences were observed in the postload IL-1*α* release between the three groups (Figures 2 and 3).

Concentrations of IL-33, IL-6, and EGF were not detectable or only detectable in very low quantities in the samples; therefore, they could not be used to assess the skin response to loading in this study.

### 3.3. Correlation of IL-1*α*, Reactive Hyperaemia, and Glucose Levels

**Figure 4:**
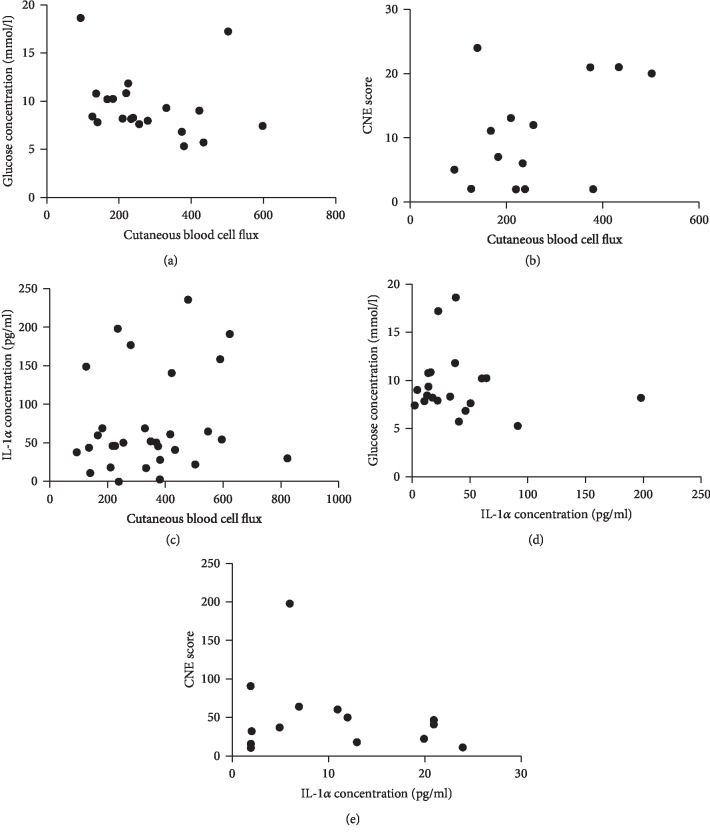
(a) Correlation between baseline glucose concentration in blood (mmol/l) and cutaneous blood cell flux directly after shear combined with pressure loading (time point 0) in patients with type 2 DM (-0.5, 95% CI -0.8 to –0.01, *P* = 0.04). Correlation was tested with the Spearman rank test. (b) Correlation between CNE score and cutaneous blood cell flux directly after shear combined with pressure loading (time point 0) in patients with a CNE score of ≥1 (0.2, 95% CI –0.4 to 0.7, *P* = 0.4). Correlation was tested with the Spearman rank test. (c) Correlation between IL-1*α* concentration (pg/ml) and cutaneous blood cell flux (AU) directly after shear combined with pressure loading (time point 0) (0.2, 95% CI -0.2 to 0.5, *P* = 0.4). Correlation was tested with Spearman rank test (*P* > 0.05). (d) Correlation between baseline glucose concentration in blood (mmol/l) and IL-1*α* concentration (pg/ml) directly after shear combined with pressure loading (time point 0) in patients with type 2 DM (-0.1, 95% CI -0.5 to 0.4, *P* = 0.6). Correlation was tested with the Spearman rank test (*P* > 0.05). (e) Correlation between VALK score and IL-1*α* concentration (pg/ml) directly after shear combined with pressure loading (time point 0) (95% CI -0.6 to 0.5, *P* = 0.7). Correlation was tested with the Spearman rank test (*P* > 0.05). Only participants with a VALK score of ≥1 were included in the analysis.

There was a negative correlation (*r* = −0.5, 95% CI -0.8 to −0.01, *P* = 0.04) between baseline glucose levels and CBF directly after pressure and shear loading in patients with type 2 DM with and without DNP (Figure 4(a)).

Not statistically significant correlations were found between the clinical neuropathy (CNE) score and CBF responses, IL-1*α* concentration and CBF, baseline glucose levels and IL-1*α* concentration, and the CNE score and IL-1*α* concentration immediately after shear combined with pressure loading (time point 0) (Figures 4(b)–4(e)).

## 4. Discussion

Increased biomechanical loading plays a pivotal role in foot ulceration in patients with type 2 DM, and although the response to various stimuli has been investigated in multiple studies, the effect of increased biomechanical loading—and in particular shear—is less well studied in DM patients. To the best of our knowledge, this study was the first to investigate the immunological and microcirculatory responses of the skin to shear (combined with pressure) loading in patients with type 2 DM with and without DNP.

In line with our previous study in healthy participants [7], the hyperaemic and immunological response of the skin to the application of shear (combined with pressure) was higher compared to the responses to pressure without shear in the three groups studied. The magnitude of loading was based on a previous study [7] where this model was validated on healthy volunteers. In this study, the shear of 5.9 kPa combined with pressure loading of 3.5 kPa for 30 minutes produced significant changes on the parameters derived from the robust measurement techniques. To our knowledge, there are no other studies that investigated the reactive hyperaemic and IL-1alpha skin response as a reaction to shear combined with pressure loading. Therefore, the same amount of pressure or shear combined with pressure was applied on the skin to the participants in our study.

The reactive hyperaemic response to shear (combined with pressure) was impaired in patients with type 2 DM in comparison to healthy volunteers but did not differ between the patients with type 2 DM with or without DNP. In contrast, no differences in the postload IL-1*α* release (a measure of tissue damage) were observed between the three groups.

Reactive hyperaemia is a physiological response that occurs as a reaction to tissue ischemia and damage in order to increase or restore oxygen and metabolic delivery and is considered to be an important response to protect the skin against ischemic skin damage [4]. The impaired microvascular response to shear (combined with pressure) in our patients with type 2 DM is in line with the abnormal vasodilator responses of the microvasculature to various noxious stimuli such as pressure loading, trauma, and heat [23, 24]. This abnormal responsiveness of the type 2 diabetic microcirculation is probably related to both impaired endothelial-dependent and endothelial-independent vasodilation [25, 26]. The effect of neuropathy is less clear and may depend on the stimuli applied. The hyperaemic response to heat is already disturbed very early in the course of the development of type 2 DM [24, 27] and is probably, at least in part, related to small fibre neuropathy [28]. The effect of large fibre neuropathy on vasodilator responses to local pressure is less well defined, but the reactive hyperaemic response to pressure was reduced in two studies in patients with DNP [6, 29]. It is interesting to note that we did not observe differences after the application of pressure alone between the patients with type 2 DM and the healthy controls nor within the two groups of type 2 DM patients with and without DNP in our study. A study performed by Petrofsky and colleagues [30] demonstrated that the postload reactive hyperaemic response was lower in patients with type 2 diabetes after 15 kPa pressure was applied on the skin in comparison to healthy volunteers. They also demonstrated a decrease in the reactive hyperaemic skin response in the periphery than the core body. Possibly, the discrepancy in results relating the effects of pressure alone could be explained by the location of the applied pressure (forearm) and the low amount of pressure applied in our study. However, only one and relatively mild pressure stimulus was applied in our study on the volar aspect of the forearm. We cannot exclude that different results could have been obtained when the measurements were performed on the plantar surface of the foot or hand or when a higher pressure was applied. But the main aim of our study was to investigate response to shear (combined with pressure) and not to pressure alone. Our data underscores the importance of shear in the development of tissue damage, given the markedly increased hyperaemic response and—to somewhat lesser extent—immunological responses compared to pressure alone. Unfortunately, although increased shear forces are thought to play an important role in the development of foot ulcers in patients with type 2 DM, these forces are currently very difficult to measure in this context.

The postload IL-1*α* release was significantly increased after the application of shear (combined with pressure) and after—to a lesser extent—pressure alone in all three groups. However, in contrast to the CBF readings, no significant differences were observed in the postload IL-1*α* release between healthy volunteers or patients with type 2 DM. IL-1*α* is an early marker of mechanically induced skin damage in the skin, because it is stored in the keratinocytes and will be rapidly released when they become injured [15, 16]. In addition, IL-1*α* is one of the most important DAMPs in the skin [31] and has an essential role in maintaining a normal skin barrier. The results of our study suggest that direct skin damage caused by shear (combined with pressure) did not differ between patients with type 2 DM with and without DNP and healthy volunteers, as the postload IL-1*α* concentration was the same in all three groups. Our data suggest that increased susceptibility of diabetic tissues to noxious stimuli might be more related to impaired vasodilator capacity of the microcirculation than to increased vulnerability per se. Interestingly, the amount of DNP did not affect the immunological or microvascular skin response after the application of shear in our study; in contrast, diabetic neuropathy did affect vasodilator response to local pressure in previous studies [20, 32]. We measured the skin responses to shear in the forearm, and therefore, we cannot exclude that different results might have been obtained when measurements were performed in the foot (which was technically not feasible). But, as recently reviewed by Fuchs and colleagues [33], the microcirculation in the feet and toes can fluctuate substantially, while the forearm and lower leg have a more stable microcirculation. In addition, as we only applied one specific shear force to the skin, it is also possible that with lower shear or shorter duration differences might have been observed. However, our data underscore the fact that the loss of the protective sensation with the inability to sense excessive loading to the skin remains a key factor in the development of diabetic foot ulcers in patients with DNP.

In earlier follow-up studies [34, 35], HbA1c levels were negatively associated with capsaicin (C-nociceptive-dependent) vasodilation or heat-induced vasodilation, measured with the laser Doppler flowmeter. In the current study, we observed a negative correlation between the blood glucose level at the start of the experiment and the reactive hyperaemic response after shear (combined with pressure) in the patients with type 2 DM. Also, in the large-scale epidemiological Maastricht study, both HbA1c and fasting blood glucose levels were associated with impaired microvascular function in the skin [36]. These data suggest that not only long-term metabolic control but also short-term metabolic perturbations can negatively affect skin microcirculatory responses to external noxious stimuli.

Our study has several limitations. Most of our patients with DNP had mild to moderate DNP based on their CNE score, and different results might have been observed if only patients with severe DNP had been included. In addition, we cannot exclude that different results could have been obtained if the measurements were performed on the plantar surface of the foot, an area that would have been more severely affected by DNP. Another important limitation is that we did take the effect of vasoactive medication that could influence the reactive hyperaemic skin response into account (e.g., beta blockers and nitrates). Finally, we used Sebutape as a noninvasive sampling method of skin cytokines. It is possible that with these techniques we were not able to detect other cytokines such as IL-33, EGF, and IL-6 because too low concentrations were obtained. This problem could possibly be solved by taking skin biopsies, which of course have other limitations.

In conclusion, the reactive hyperaemic skin response to shear (combined with pressure) was decreased in patients with type 2 DM. Moreover, this reactive hyperaemic skin response was negatively correlated with the ambient blood glucose value. In contrast, postload IL-1*α* release, presumably a measure of skin damage and vulnerability, was not altered in patients with type 2 DM. These data suggest that in type 2 DM in particular the reparative (microcirculatory) responses to noxious stimuli are impaired, resulting in impaired damage control once the skin has been abnormally loaded. DNP was not associated with altered skin responses to shear, suggesting that it is in particular the loss of sensation that puts patients with DNP at risk for developing tissue damage.

## Figures and Tables

**Table 1:** Baseline characteristics of the participants (*n* = 30).

	DM PNP- (*n* = 10)	DM PNP+ (*n* = 10)	Control (*n* = 10)	*P* value
Age (years)	61.0 ± 6.4	65.2 ± 4.8	64.5 ± 4.1	NS
Males (no) (%)	6 (60.0)	6 (60.0)	5 (50.0)	NS
Length	168.5 ± 9.9	172.6 ± 11.6	171.8 ± 10.5	NS
Weight	79.2 ± 15.9	86.1 ± 17.6	80.6 ± 12.6	NS
BMI (kg/m^2^)	27.9 ± 3.7	28.6 ± 2.9	27.2 ± 2.9	NS
Blood pressure (systolic)	143.9 ± 14.5	149.2 ± 20.0	139.5 ± 16.7	NS
Blood pressure (diastolic)	80.0 ± 5.7	81.0 ± 10.8	81.3 ± 11.1	NS
VALK score: median (IQR)	1.0 (0-2)	12.5 (6.8-21.0)	0 (0-0)	Yes^∗^
Glucose (mmol/l)	8.9 ± 1.9	10.1 ± 4.3	5.7 ± 1.1	Yes^∗∗^
HbA1c (%)	8.2 ± 1.0	7.6 ± 0.9	N.D.	NS
Creatinine	80.5 ± 17.3	83.3 ± 14.8	76.3 ± 15.5	NS

DM PNP- indicates the diabetes mellitus without polyneuropathy group. DM PNP+ indicates the diabetes mellitus with polyneuropathy group. ^∗^DM PNP- vs. control (*P* < 0.05) and DM PNP+ vs. control (*P* < 0.01). ^∗∗^DM PNP- vs. DM PNP+ (*P* < 0.01) and DM PNP- vs. control (*P* < 0.001).

## Data Availability

The data used to support the findings of this study are available from the corresponding author upon request.
